# Inadequate Processing of Decellularized Dermal Matrix Reduces Cell Viability *In Vitro* and Increases Apoptosis and Acute Inflammation *In Vivo*

**DOI:** 10.1089/biores.2016.0021

**Published:** 2016-07-01

**Authors:** Aaron H. Morris, Julie Chang, Themis R. Kyriakides

**Affiliations:** ^1^ Department of Biomedical Engineering, Yale University, New Haven, Connecticut.; ^2^ Department of Vascular Biology and Therapeutics Program, Yale University, New Haven, Connecticut.; ^3^ Department of Pathology, Yale University, New Haven, Connecticut.

**Keywords:** biomaterials, extracellular matrix, inflammation, tissue engineering

## Abstract

Decellularized tissue scaffolds are commonly used in the clinic because they can be used as substitutes for more traditional biomaterials, while imparting additional physiological effects. Nevertheless, reports of complications associated with their use are widespread and poorly understood. This study probes possible causes of these complications by examining cell viability and apoptosis in response to eluents from decellularized dermis. Using multiple sources of decellularized dermis, this study shows that typical decellularized scaffolds (prepared with commonly used laboratory techniques, as well as purchased from commercial sources) contain soluble components that are cytotoxic and that these components can be removed by extensive washes in cell culture media. In addition, this study demonstrates that these observed *in vitro* phenotypes correlate with increased apoptosis and acute inflammation when implanted subcutaneously in mice.

## Introduction

Decellularized tissue scaffolds are frequently used in a variety of clinical applications such as chronic wound coverings, hernia repair, and heart valve replacements.^1–3^ The use of decellularized products has expanded rapidly because they can function as substitutes for traditional biomaterials (e.g., polyurethanes, PLGA, etc.) while offering the additional advantage of retaining native extracellular matrix (ECM) structure. Thus, they can serve as inductive scaffolds for cell invasion. In addition to their structural similarity to native tissue, decellularized scaffolds are effective because they can incorporate matrix-bound growth factors and other bioactive molecules that can be released upon degradation.^4–6^ Current commercially available decellularized biologic scaffolds are derived from several tissue sources, including skin, small intestinal submucosa, pericardium, and bladder.

Scaffolds are prepared in numerous ways, many of which are proprietary. Generally, decellularization methods fall into three broad categories: physical, chemical, and enzymatic.^4,7–11^ Most scaffolds are prepared using a combination of these methods, the most popular of which is chemical and enzymatic.^8,12^ Decellularization methods are tissue specific; for example, decellularization of thicker tissues often requires the perfusion of decellularization agents, rather than simple washes. Regardless of the decellularization method, the terminal steps almost always include extensive washing, usually with phosphate-buffered saline (PBS) or deionized water. Furthermore, scaffolds must either be decontaminated before decellularization and, subsequently, handled aseptically or terminally sterilized using ethylene oxide, ultraviolet irradiation, gamma irradiation, or supercritical carbon dioxide.^8,13–16^ Finally, the scaffolds are prepared for storage and shipment, which can include packaging of hydrated matrix or lyophilizing to dry and preserve the matrix before packaging.

Techniques used to characterize prepared scaffolds include the following: histological stains to detect cellular and nuclear remnants, as well as to visualize ECM architecture; quantification of residual DNA and nuclear fragments; electron microscopy; mechanical testing; and other material characterization assays.^9–12,17–19^ Tests for material characterization often include direct cell contact on the material of interest or indirect assays in which the material of interest is incubated in cell media at a defined concentration—any soluble components of the material of interest are eluted into the media to create a liquid extract, which is in turn evaluated for effects on cultured cells.^20–23^ The effects of the eluent on cells can be examined using viability or proliferation assays, as well as various functional tests. Subsequent *in vivo* tests often include subcutaneous implantation to examine the *in vivo* toleration and host response to the material.^18,24,25^

Despite the aforementioned advantages of decellularized scaffolds, reports of complications are widespread. In many applications, including tissue expander breast reconstruction, use of decellularized biological scaffolds significantly increased the complication rate relative to the use of synthetic scaffolds.^26–28^ Common complications documented include infection, dehiscence, skin necrosis, and seroma;^26–31^ yet, the cause of these complications is currently unknown and has been difficult to determine. Generally, decellularized scaffolds are cytocompatible and exhibit a constructive reparative phenotype upon implantation.^4,22^ Investigations into ineffective decellularization techniques reveal that materials that contain significant residual DNA exhibit a pro-inflammatory response.^31–33^ In addition, a small, but growing, number of investigations have shown that decellularized scaffolds may have inhibitory effects on cell proliferation, or even exhibit cytotoxic effects.^16,21,34–40^ Investigators have attributed the causes of these negative effects to a variety of factors, such as residual detergents, residual sterilization chemicals, and alterations of matrix structure or biochemistry due to decellularization.^16,35,37^

Herein we investigated the cytocompatibility of decellularized scaffolds sourced from mouse skin decellularized with several methods and AlloDerm, a commercially available human dermis product. We used elution assays to study the response of keratinocytes and fibroblasts to these materials and investigated the cellular responses using metabolic viability and apoptosis assays. Because of a paucity of data regarding the ideal mass of matrix necessary for these tests, we tested a range of soluble fraction dilutions. In addition, we examined the host response to these materials by subcutaneous implantation in mice. We demonstrate that acellular dermal materials can, at sufficiently high doses, lead to apoptosis both *in vitro* and *in vivo*, but that additional washes beyond the standard protocols mitigate this phenotype.

## Materials and Methods

### Isolation of murine skin

All procedures were performed in accordance with the regulations adopted by the National Institutes of Health and approved by the Animal Care and Use Committee of Yale University. Immediately following euthanasia, 12–14-week-old C57BL/6 mice were shaved and treated with depilatory cream for 1 min to remove remaining hair. Entire skins were subsequently harvested from the dorsum and flash frozen in liquid nitrogen. Frozen skins were stored at −80°C until use.

### Decellularization with trypsin and Triton

Frozen skins were thawed, and the epidermal side was gently scraped with a scalpel to remove the stratum corneum of murine skins. Skins were then treated with a protocol developed by Reing et al. for porcine skin.^12^ Briefly, skins were incubated for 6 h in 0.25% Trypsin-EDTA (J.T. Baker), followed by washes in ddH_2_0 thrice for 15 min. Skins were incubated in 70% ethanol for 12 h and 3% H_2_O_2_ (Sigma) for 15 min, followed by two 15-min washes in ddH_2_0. Skins were then incubated in 1% Triton X-100 (American Bioanalytical) in 0.26% Tris (American Bioanalytical)/0.69% EDTA (Sigma) for 6 h and then overnight. Finally, skins were washed in ddH_2_0 six times for 15 min each. All above steps were performed at room temperature on an orbital shaker. Samples prepared in this manner will be referred to as d-TT (decellularized with trypsin and Triton). Skins receiving an extended wash were washed in serum-free Dulbecco's modified Eagle's medium (DMEM; Gibco) with 1% penicillin/streptomycin (Pen Strep; Gibco) on a rotating shaker at 37°C for 24 h. These samples will be referred to as d-TT EW (decellularized with trypsin and Triton extended wash). Afterward, skins were lyophilized and stored in a desiccator until use. Samples of each skin were fixed, prepared for histological analysis, and then stained with hematoxylin and eosin using standard protocols. To grind decellularized skin, the lyophilized skin was cut into 1 cm wide strips and placed into the grinding chamber of a Porlex Mini Coffee Grinder (Amazon no. B0044ZA066) with the burr set to the finest grind. The skin was then ground until all of the skin became particulate matter. For scanning electron microscopy (SEM), the powder was adhered to carbon tape on top of an SEM stub. The powder was then chrome coated with ∼20 nm of chrome and imaged with a Hitachi SU-70 SEM.

### Decellularization with NaOH

To perform detergent-free decellularization, a previously published protocol was modified for use on mouse skin.^41^ Frozen skins were thawed and placed in 0.25% Trypsin-EDTA at 37°C for 90 min. All subsequent steps were performed at room temperature on an orbital shaker. Skins were washed in ddH_2_0 thrice for 15 min and then incubated in 0.1 M NaOH (Macron) for 16 h. The skins were then washed in Dulbecco's phosphate-buffered saline (DPBS; Gibco) until the pH of the rinsing solution was neutral. If the skins received an extended wash, they were washed in serum-free DMEM with 1% pen strep on a rotating shaker at 37°C for 24 h. Skins were lyophilized and stored in a desiccator until use. Samples of each skin were fixed and prepared for histological analysis and then stained with hematoxylin and eosin using standard protocols.

### Preparation of liquid extracts from matrix

AlloDerm was a generous gift from Dr. Deepak Narayan (Department of Surgery, Yale University).

Liquid extracts were prepared using a method similar to those set by the International Organization for Standardization (ISO 10993-5:2009). It is important to note that many previously conducted extract cytotoxicity experiments only tested one concentration, but the ISO recommends testing a range of concentrations with an upper bound of 0.1 g material per mL of fluid. Many prior studies use 1 mg/mL of material or less and also do not study a range of concentrations.^9,42–44^ To perform a more comprehensive study, we prepared an extract by incubating 20 mg of lyophilized ECM per mL of media, which was further diluted to generate a range of extracts (representing the soluble fraction from 0.25 to 10 mg/mL). To prepare the original extract, a known mass of lyophilized matrix was sterilized by UV for 15 min and then added to serum-free DMEM with 1% pen strep to reach 20 mg/mL. This incubation was performed on a rotating shaker at 37°C for 24 h. Separate medium was prepared in parallel to serve both as the control and as the dilution medium for the original extracts. Dilutions of the original extract were performed to generate samples representing decreasing amounts of ECM (10, 4, 2, 1, 0.5, and 0.25 mg/mL). The extracts were then centrifuged at 2773 *g* for 10 min to remove any large particulate matter. Fetal bovine serum (FBS) was added to the media to a concentration of 10% FBS.

### Cell culture

Mouse embryonic fibroblast cell line NIH3T3 (ATCC) was chosen as the model fibroblast cell line for the media extract experiments. Mouse keratinocyte cell line PAM212 was a generous gift from the laboratory of Dr. Gunter Wagner (Department of Ecology and Evolutionary Biology, Yale University). Cells were maintained in growth medium, DMEM, with 10% FBS and 1% pen strep.

### Extract viability assay

Cells were harvested by addition of 0.25% Trypsin/EDTA. A suspension of 5000 cells was added to each well of a 96-well plate and allowed to adhere overnight at 37°C and 5% CO_2_. Media was then removed and replaced with extract medium, and the cells were incubated for 24 h at 37°C and 5% CO_2_. Extract media was removed, replaced with normal growth media, and cell viability was determined using the CellTiter-Blue assay (Promega), according to manufacturer's instructions.

### TUNEL assay

Cells were harvested by addition of 0.25% Trypsin/EDTA. A suspension of 5000 cells was added to each well of a 96-well plate and allowed to adhere overnight at 37°C and 5% CO_2_. Media was then removed and replaced with extract medium, and the cells were incubated for 1 h at 37°C and 5% CO_2_. Cells were fixed for 1 h in 4% PFA (J.T. Baker). TUNEL staining was performed using the *In Situ* Cell Death Detection Kit, POD from Roche (catalog no. 11684817910), according to the manufacturer's instructions. The Converter-POD was not used; instead, the cells were mounted with VECTASHIELD containing DAPI (Vector Labs) and imaged using fluorescence microscopy. The number of TUNEL-positive cells per high power field (20 ×) was quantified using three fields per well. Three repeat experiments were performed (*n* = 3), and all experiments were analyzed by the same person.

### Subcutaneous implantation

All procedures were performed in accordance with the regulations adopted by the National Institutes of Health and approved by the Animal Care and Use Committee of Yale University. Using a biopsy punch, 4 mm diameter implants were punched from lyophilized decellularized skins and then sterilized with UV irradiation. The thickness was determined by the original thickness of the mouse skin, but averaged 0.59 mm. Subcutaneous implantations were performed as previously described.^45^ A total of eight C57BL/6 mice were used, four for each time point: 3 and 14 days. Each mouse received two subcutaneous implants in its dorsal region, one d-TT and one d-TT EW. Implants were excised en bloc upon termination of the study and prepared for histological analysis. Sections were stained with hematoxylin and eosin according to standard protocols. In addition, samples were analyzed by immunohistochemistry, with an antibody against macrophage antigen-3 (Mac-3) (BD Pharmingen), an antibody against mouse Ly-6B (a neutrophil marker; Serotec), and with a TUNEL assay (*In Situ* Cell Death Detection Kit, POD from Roche [catalog no. 11684817910]). The slides were imaged and the number of Ly-6B, Mac-3, or TUNEL-positive cells per high power field (20 ×) was quantified using three fields per implant.

### Statistical analysis

Data are expressed as the mean + the standard error of the mean. A two-tailed Student's *t*-test was used, and *p*-values <0.05 were considered statistically significant.

## Results

### Decellularization and processing

**FIG. 1.**
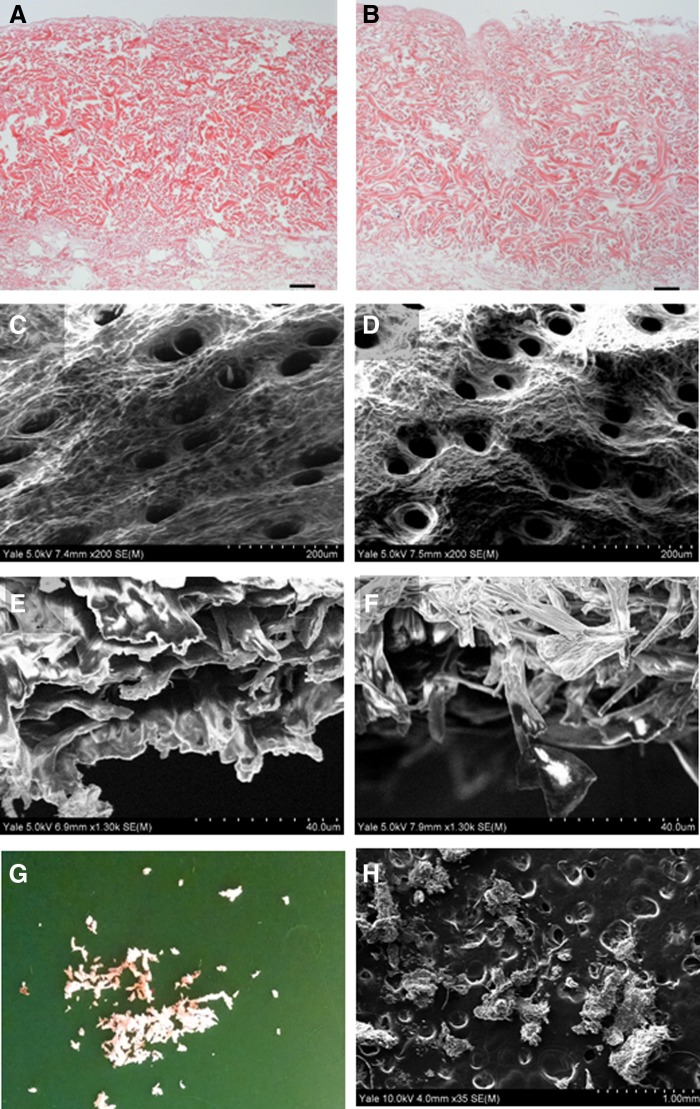
H&E of d-TT **(A)** and d-TT EW **(B)** indicating the absence of nuclear material in decellularized skin (scale bar is 50 μm). SEM micrographs of the epidermal side of d-TT **(C)** and d-TT EW **(D)**. SEM of the cross section of d-TT **(E)** and d-TT EW **(F)**. Gross appearance of particulate d-TT skin after grinding **(G)** and SEM micrograph of the same **(H)** indicating a particle diameter of ∼350 μm. d-TT, decellularized with trypsin and Triton; d-TT EW, decellularized with trypsin and Triton extended wash; H&E, hematoxylin & eosin.

The aim of the d-TT decellularization process was to adequately remove cellular components while retaining the native dermal architecture of mouse skin. Although a similar process had previously been used to decellularize porcine skin, it has never been used on mouse skin to our knowledge.^12^ Decellularization resulted in adequate removal of cellular and nuclear material, which was clearly demonstrated by the lack of nuclear remnants on the hematoxylin and eosin stained tissue (Fig. 1B). d-TT and d-TT EW retained dermal structure similar to that observed in native skin (Fig. 1A–F). Subsequent grinding of the skin produced irregular particulate matter with diameters of ∼350 μm (Fig. 1G, H).

### Cytotoxicity testing of d-TT skin

**FIG. 2.**
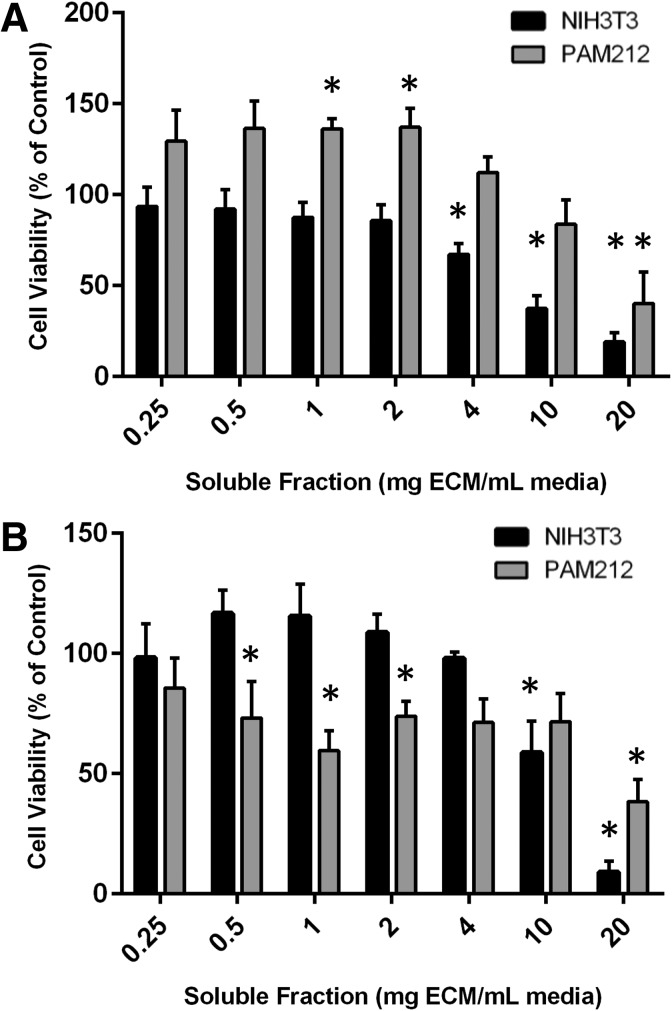
Results of a CellTiter-Blue assay indicate that d-TT extract exhibited no effects on NIH3T3 cell viability (black bars) at dilutions representing 0.25–2 mg matrix/mL media, but decreased the amount of viable cells at dilutions representing 4 mg/mL and above compared to 0 mg/mL. d-TT extract did not affect PAM212 cells at 0.25, 0.5, 4, or 10 mg/mL; slightly increased the cell viability at 1 and 2 mg/mL; and decreased cell viability at 20 mg/mL (gray bars). **(A)** Extracts of the commercially available reconstructive material AlloDerm had no effects on NIH3T3 cells at dilutions representing 0.25–4 mg/mL, but reduced the amount of viable cells at 10 and 20 mg/mL compared to the 0 mg/mL control. AlloDerm had no effects on PAM212 cell viability at dilutions representing 0.25, 4, or 10 mg/mL and decreased cell viability at 0.5, 1, 2, and 20 mg/mL. **(B)** *p < 0.05.

Soluble components of the lyophilized ECM were extracted into serum-free DMEM supplemented with 1% pen strep. Serum was added to a final concentration of 10%, and the resulting solutions were placed on adherent cells. With soluble fractions from <2 mg matrix/mL media (similar to those that are frequently tested in the literature), either no effects or a slight increase in viable cells was observed with both NIH3T3 fibroblast cells and PAM212 keratinocytes. Interestingly, with increasing soluble fractions (>4 mg/mL in the case of NIH3T3s or 20 mg/mL in PAM212s), significantly fewer viable cells were observed in the d-TT extracted medium (Fig. 2A). The effects of the particulate matrix were similar to those of the intact slabs (Supplementary Fig. S1). Boiling the material extracts or treating them with trypsin (before the addition of serum) did not mitigate the toxicity (Supplementary Fig. S2).

### Cytotoxicity testing of AlloDerm

AlloDerm, a commercially available decellularized skin scaffold, was obtained and used as a point of comparison for the d-TT matrix. Material extract prepared from AlloDerm in sufficiently high doses also reduced the number of viable cells compared to control. NIH3T3 cells seemed more resistant to the effects of AlloDerm compared to the murine d-TT, with significantly fewer cells in only the 10 and 20 mg/mL groups. PAM212 cells were more sensitive to the AlloDerm extract compared to the murine d-TT, with significantly fewer cells even in the 0.5 mg/mL group. It is worth noting that the cell viability was well above 50% at concentrations below 20 mg/mL (Fig. 2B).

### Cytotoxicity testing of d-TT EW

**FIG. 3.**
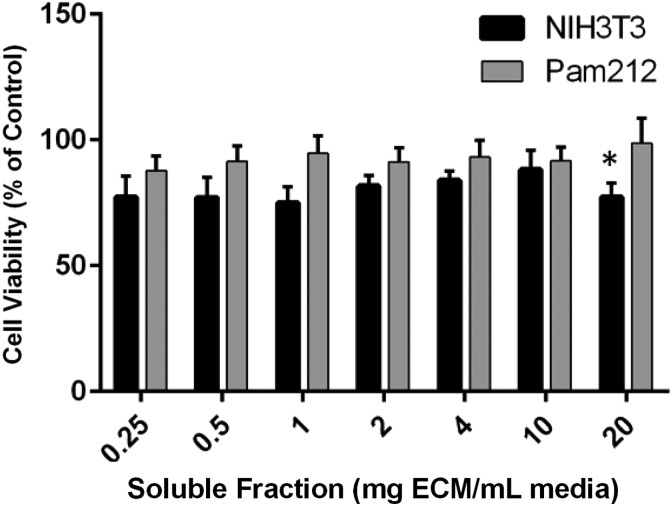
Results of a CellTiter-Blue assay indicate that an extended wash of d-TT skin at 37°C for 24 h rescued the negative effects that d-TT skin has on NIH3T3 (black bars) and PAM212 (gray bars) cells. With a soluble fraction prepared from 20 mg/mL, the extract resulted in a slight, but significant reduction in the amount of viable NIH3T3s. *p < 0.05.

Because the reduction in viability by matrix products in Figure 2 was observed with extracts produced by incubating them in media, we investigated whether washing the matrix in serum-free cell media at 37°C for 24 h would help mitigate the reduction in viability. When d-TT received this extended wash during its initial processing, it no longer had any negative effects on the viability of PAM212s and showed a slight, but significant, reduction in viability of the NIH3T3s in only the 20 mg/mL soluble fraction. Interestingly, the slight increase in PAM212 cell viability in the d-TT also disappeared with this extended wash (Fig. 3).

### Cytotoxicity testing of NaOH decellularized skins

**FIG. 4.**
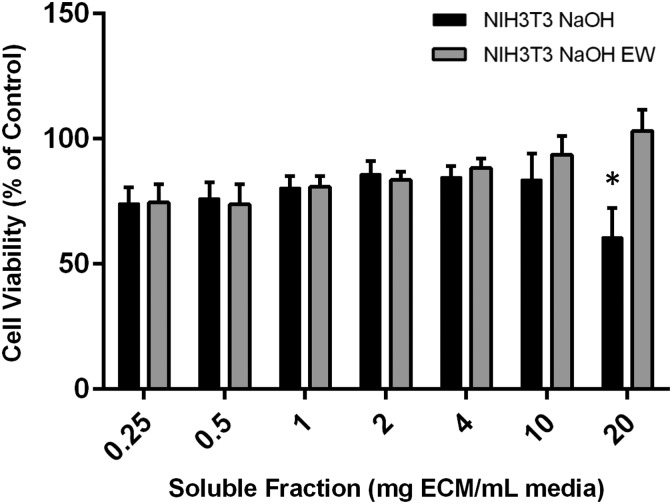
Results of a CellTiter-Blue assay indicate that extracts of skin decellularized with 0.1 M NaOH for 16 h significantly reduced the amount of viable NIH3T3 cells at 20 mg/mL (black bars), but that an extended wash of the decellularized skins at 37°C for 24 h rescued the phenotype (gray bars). *p < 0.05.

To address the possibility of residual detergents remaining in the decellularized matrix, another set of decellularized skins was prepared using 0.1 M NaOH as the decellularization reagent instead of the d-TT protocol. Upon buffering, NaOH should be rendered harmless such that any residual decellularization solution will simply become salt. Similar to d-TT, NaOH decellularized skins significantly reduced viability of NIH3T3s at 20 mg/mL. Moreover, an extended wash in media mitigated this effect (Fig. 4). This effect was not seen in PAM212 cells (Supplementary Fig. S3).

### Apoptotic response to extracts

**FIG. 5.**
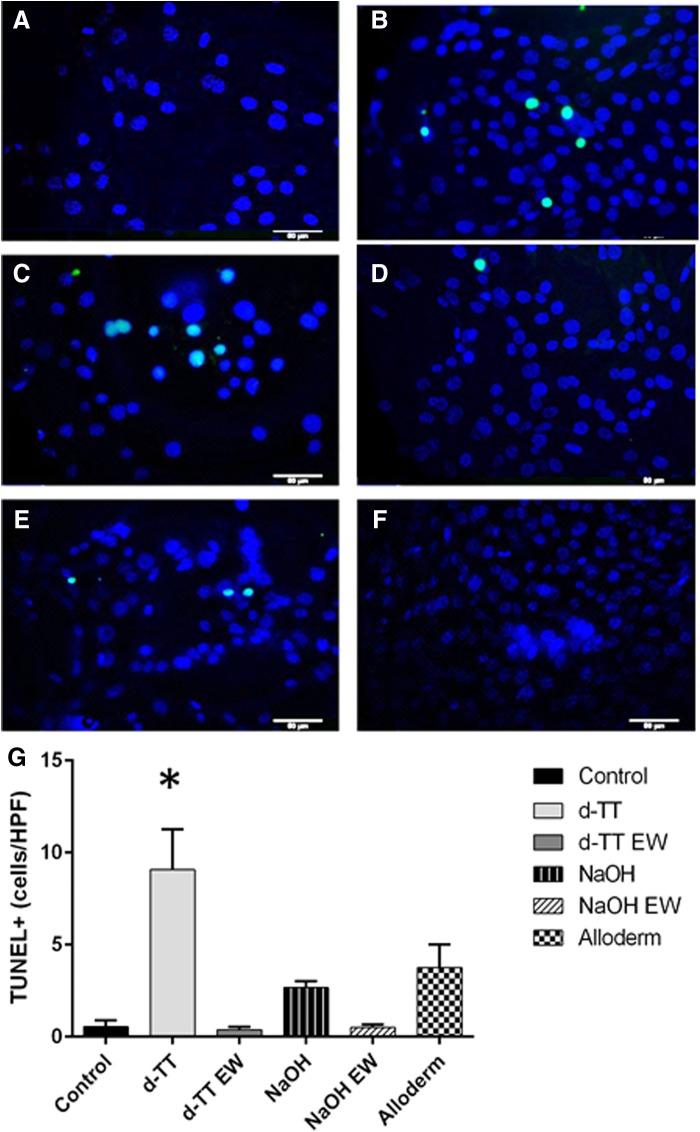
TUNEL stain (green) and DAPI (blue) of NIH3T3 cells after a 1-h incubation in 20 mg/mL extracts from the following materials: untreated control **(A)**, AlloDerm **(B)**, d-TT **(C)**, d-TT EW **(D)**, NaOH decellularized skin **(E)**, and NaOH decellularized EW **(F)** (scale bar is 50 μm). Quantification of the number of TUNEL-positive cells per HPF indicates that d-TT extracts caused significantly more apoptosis at 1 h compared to control **(G)**. *p < 0.05. HPF, high power field.

To investigate the reduction in cell viability caused by the addition of ECM extracts, adherent NIH3T3 cells were treated with 20 mg/mL extracts for 1 h at 37°C, fixed, and analyzed with the *In Situ* Cell Death Detection Kit, POD from Roche. While apoptotic cells were observed in each material extract, d-TT induced more apoptosis (Fig. 5).

### *In vivo* implantation

**FIG. 6.**
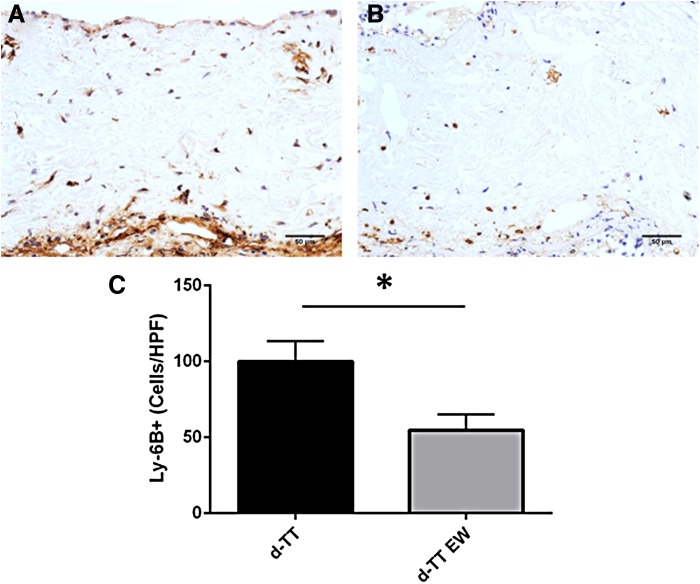
Sample d-TT **(A)** and d-TT EW **(B)** were implanted subcutaneously for 3 days before excision and immunohistochemical analysis. d-TT showed increased Ly-6B-positive cells (neutrophils) compared to d-TT EW (scale bar is 50 μm). Quantification of staining **(C)** shows increases in Ly-6B in d-TT. *p < 0.05.

**FIG. 7.**
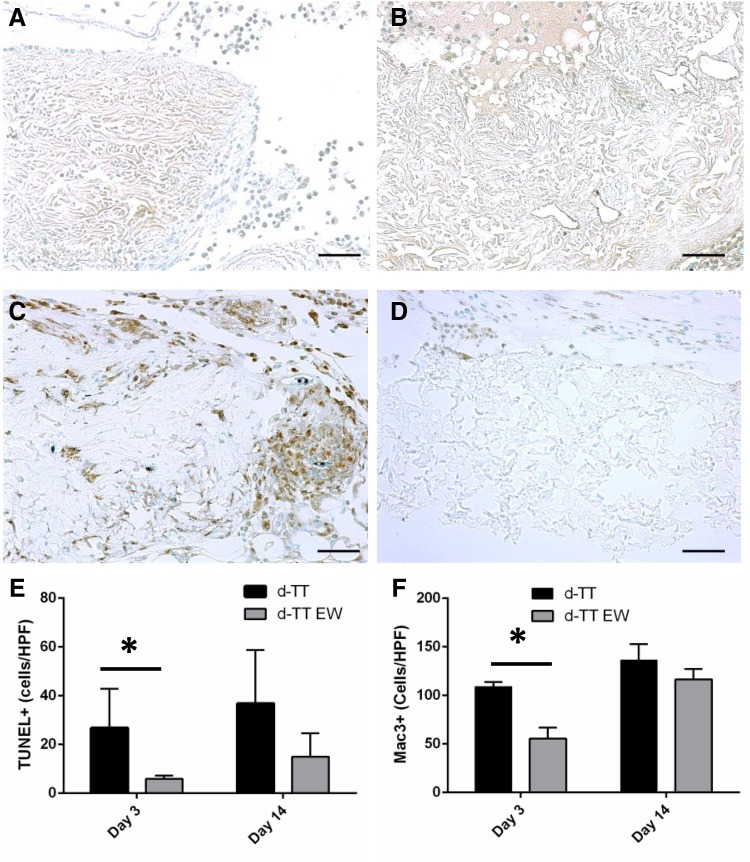
Samples d-TT **(A, C)** and d-TT EW **(B, D)** were implanted subcutaneously for 3 **(A–D)** and 14 days before excision and immunohistochemical analysis. d-TT showed increased TUNEL-positive cells **(A)** and Mac-3-positive cells **(C)** at 3 days compared to d-TT EW (**B, D,** respectively) (scale bar is 50 μm). Quantification of TUNEL staining **(E)** and Mac-3 staining **(F)** shows increases in these stains in d-TT at day 3, but no difference between groups at day 14. *p < 0.05.

To determine any physiological effects of the observed extract toxicity, 4 mm disks of the lyophilized d-TT or d-TT EW materials were implanted subcutaneously in C57/BL6 mice for 3 and 14 days. Each mouse received two implants, one d-TT and one d-TT EW. Upon removal, each material was embedded and prepared for immunohistochemistry. H&E staining showed no gross difference between d-TT and d-TT EW (Supplementary Fig. S4). Ly-6B staining for neutrophils showed increased neutrophil presence in the d-TT implants at day 3 compared to d-TT EW (Fig. 6); by day 14, the neutrophil presence was too low to quantify (data not shown). TUNEL staining showed more apoptotic cells around the implants in d-TT scaffolds compared to d-TT EW at day 3, while by day 14 there was no difference between the groups (Fig. 7A, B, E). In addition, Mac-3 staining showed increased macrophage numbers around the d-TT implants at day 3, but by day 14 there was no difference (Fig. 7C, D, F).

## Discussion

This study investigated the cytocompatibility of materials derived from decellularized skin of murine and human origin. Routine extract cytotoxicity assays were the principle method used to study the materials because of their ubiquitous use among the biomaterial community. Interestingly, we found that mouse skin decellularized with techniques commonly used for porcine skin retained soluble factors that caused cytotoxicity in sufficiently high doses in a dose-dependent manner.^12^ These results were initially surprising because most reports of extract cytotoxicity testing of decellularized ECM materials reported no toxicity and often reported positive cellular effects.^9,22,23^ Nevertheless, upon closer examination of the literature, we discovered that there are a growing number of reports indicating that decellularized matrix materials inhibit proliferation, enhance the apoptotic response, or even cause toxicity.^16,21,34–40^ We also observed that the soluble factors causing cytotoxicity were not rendered inert by boiling or treating the extract medium with trypsin.

Furthermore, we determined that AlloDerm caused similar dose-dependent toxicity. An additional washing step (in DMEM for 24 h at 37°C) rescued the toxic effects of the scaffolds. The toxicity was most likely caused by residual detergent on the materials (despite lengthy washing procedures). To investigate this hypothesis, we decellularized skins with a detergent-free sodium hydroxide method. Because no detergents are used in this method, there cannot be any residual decellularization solutions upon the buffering of the base. Yet, the NaOH decellularized skins still exhibited a toxic effect on NIH3T3 cells. This effect was not as pronounced as that observed for d-TT decellularized skins or AlloDerm, but it remained nonetheless. Moreover, extended washing of the NaOH decellularized skins rescued the toxicity for these samples as well. This suggests that although residual detergents may play a role in the observed toxicity, they cannot be the only cause. We conclude that decellularized ECM contains other factors that cause cell toxicity. In fact, we determined that the d-TT skins induced apoptosis even at the early time point of 1 h.

To investigate whether these observations correlate with complications *in vivo,* we implanted disks of lyophilized decellularized skins subcutaneously in mice for 3 and 14 days. We observed that the d-TT skins caused more apoptosis, as well as neutrophil and macrophage recruitment, compared to the d-TT EW at 3 days. This suggests that the soluble cytotoxic factors observed in these ECM materials *in vitro* lead to increased early apoptosis and acute inflammation *in vivo*. Because of the increasing number of reports of complications when using decellularized ECM materials in a surgical setting, these results could help us better understand the complicated host response to these materials. Further research into these materials and what is causing these effects is needed to create solutions for surgical complications.

## Supplementary Material

Supplemental data

## Abbreviations Used

DMEM: Dulbecco's modified Eagle's medium
d-TT: decellularized with trypsin and Triton
d-TT EW: decellularized with trypsin and Triton extended wash
ECM: extracellular matrix
H&E: hematoxylin & eosin
PBS: phosphate-buffered saline
SEM: scanning electron microscopy
