# Supplementary material for: Inadequate Processing of Decellularized Dermal Matrix Reduces Cell Viability In Vitro and Increases Apoptosis and Acute Inflammation In Vivo
Source: Biores Open Access. 2016 Jul 1;5(1):177–87. doi: 10.1089/biores.2016.0021 (PMC4948200; doi:10.1089/biores.2016.0021)
Supplement: Supplemental data [file Suppl_Data.pdf]

Supplementary Data

Preparation of Boiled Extracts

Extracts were prepared from d-TT as described in the Materials and Methods section and, subsequently, boiled for 10 min before cooling the samples on ice and adding serum. These samples were then placed onto cells that were seeded as described in the Materials and Methods section. Only soluble fractions of 10 and 20 mg/mL were used in the boiled extract experiments.

Preparation of Trypsin-Treated Extracts

Extracts were prepared from d-TT as described in the Materials and Methods section, and trypsin from porcine pancreas (Sigma) was subsequently added to reach a concentration of 0.0625%. The samples were incubated at 37°C for 15 min and the trypsin was neutralized by addition of 10% FBS. These samples were then placed onto cells that were seeded as described in the Materials and Methods section. Only soluble fractions of 10 and 20 mg/mL were used in the trypsin-treated extract experiments.

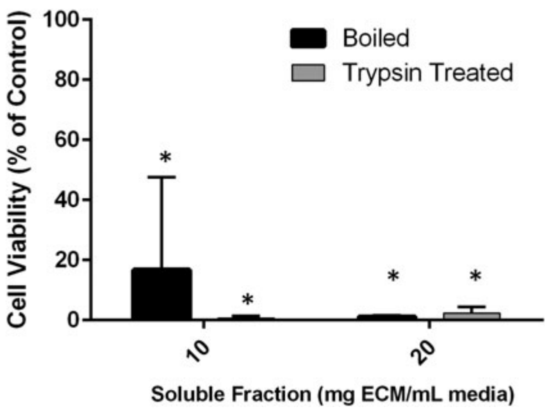

SUPPLEMENTARY FIG. S2. Neither boiling (black bars) nor trypsin treatment (gray bars) of the d-TT extracts was able to mitigate the reduction in viability of NIH3T3 cells at dilutions representing 10 or 20 mg/mL compared to 0 mg/mL control. *$p < 0.05$.

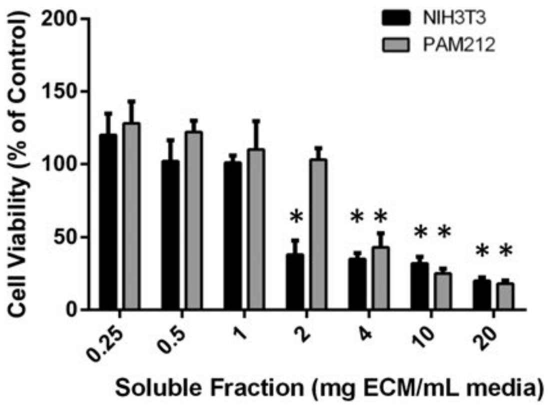

SUPPLEMENTARY FIG. S1. Results of a CellTiter-Blue assay indicate that extract from a particulate form of the d-TT matrix exhibited no effects on NIH3T3 cells at dilutions representing 0.25–1 mg/mL, but decreased the amount of viable cells at 2 mg/mL and above compared to 0 mg/mL (black bars). Particulate d-TT extract did not affect PAM212 cells at 0–2 mg/mL, but decreased the cell viability at dilutions representing 4 mg/mL and above (gray bars). *$p < 0.05$.

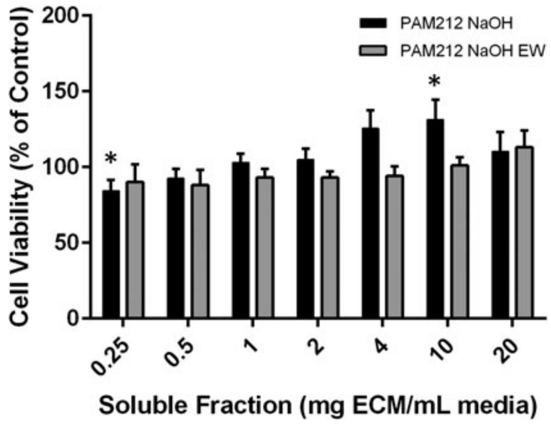

SUPPLEMENTARY FIG. S3. Results of a CellTiter-Blue assay indicate that extracts of skin decellularized with 0.1 M NaOH for 16 h slightly reduced the number of viable PAM212s at 0.25 mg/mL and increased the viable number of cells at 10 mg/mL. It had no effects with other dilutions or after the decellularized skins received an extended wash. *$p < 0.05$.

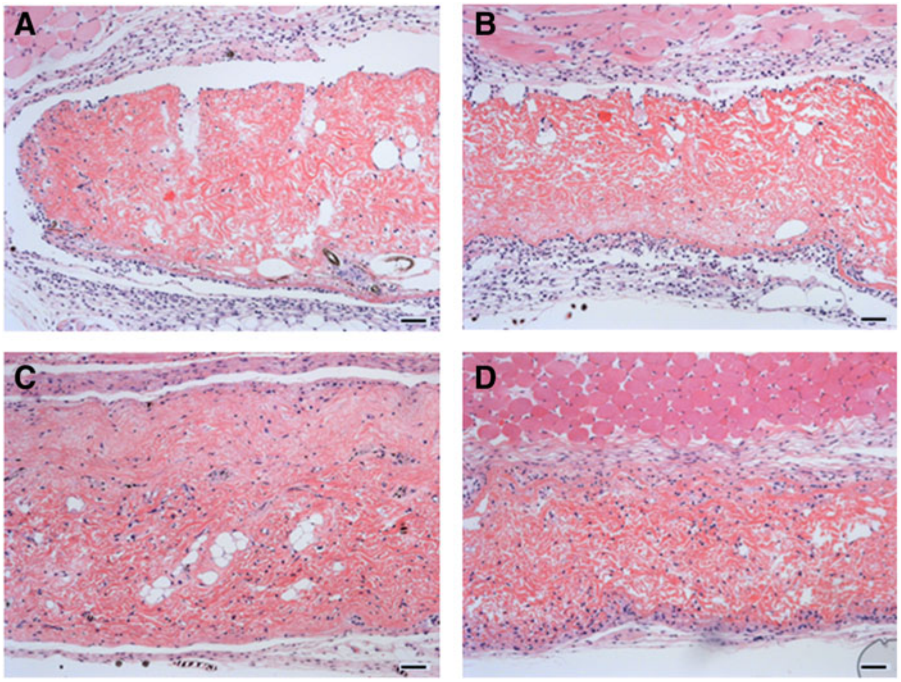

SUPPLEMENTARY FIG. S4. Samples d-TT **(A, C)** and d-TT EW **(B, D)** were implanted subcutaneously for 3 **(A, B)** and 14 days **(C, D)** prior to excision and staining with hematoxylin and eosin.
